# Pharmacokinetic analysis and simplified uptake measures for tumour lesion [^18^F]F-AraG PET imaging in patients with non-small cell lung cancer

**DOI:** 10.1007/s00259-024-06931-3

**Published:** 2024-10-08

**Authors:** Jessica E. Wijngaarden, Maarten Slebe, Johanna E. E. Pouw, Daniela E. Oprea-Lager, Robert C. Schuit, Chris Dickhoff, Jelena Levi, Albert D. Windhorst, C. Willemien Menke-van der Houven van Oordt, Andrea Thiele, Idris Bahce, Ronald Boellaard, Maqsood Yaqub

**Affiliations:** 1 Department of Radiology and Nuclear Medicine, Amsterdam UMC Location Vrije Universiteit Amsterdam, Amsterdam, Netherlands; 2 Cancer Center Amsterdam, Imaging and Biomarkers, Amsterdam, The Netherlands; 3 Department of Pulmonary Medicine, Amsterdam UMC Location Vrije Universiteit Amsterdam, Amsterdam, The Netherlands; 4 Department of Medical Oncology, Amsterdam UMC Location Vrije Universiteit Amsterdam, Amsterdam, The Netherlands; 5 Department of Cardiothoracic Surgery, Amsterdam UMC Location Vrije Universiteit Amsterdam, Amsterdam, The Netherlands; 6 CellSight Technologies Incorporated, San Francisco, CA USA; 7 Department of Translational Medicine & Clinical Pharmacology, Boehringer Ingelheim Pharma GmbH & Co. KG, Biberach an der Riss, Germany

**Keywords:** [^18^F]F-AraG, ImmunoPET, Pharmacokinetic modelling, Activated T-cells

## Abstract

**Introduction:**

The novel positron emission tomography (PET) imaging tracer, [^18^F]F-AraG, targets activated T-cells, offering a potential means to improve our understanding of immune-oncological processes. The aim of this study was to determine the optimal pharmacokinetic model to quantify tumour lesion [^18^F]F-AraG uptake in patients with non-small cell lung cancer (NSCLC), and to validate simplified measures at different time intervals against the pharmacokinetic uptake parameter.

**Methods:**

Ten patients with early-stage NSCLC and three patients with advanced NSCLC underwent a dynamic PET scan of minimal 60 min. Venous and/or arterial blood sampling was obtained at maximum seven time points. Tumour lesion time activity curves and metabolite-corrected input functions were analysed using single-tissue reversible (1T2k), two-tissue irreversible (2T3k) and two-tissue reversible (2T4k) plasma input models. Simplified uptake measures, such as standardised uptake value (SUV) and tumour-to-blood (TBR) or tumour-to-plasma ratio (TPR), were evaluated for different time intervals.

**Results:**

Whole-blood and plasma radioactivity concentrations showed rapid clearance of [^18^F]F-AraG. Metabolite analysis revealed a low rate of metabolism, at 70 min p.i., on average, 79% (SD = 9.8%) of the total radioactivity found in blood corresponded to intact [^18^F]F-AraG. The time activity curves were best fitted by the 2T3k model. Strong positive correlations were found for SUV (body weight (BW), lean body mass (LBM) or body surface area (BSA) corrected), TBR and TPR for any time interval between 20 and 70 min p.i. against the 2T3k-derived *K*_*i*_. The correlation of TBR at 60–70 min p.i. with 2T3K-derived *K*_*i*_ (r (df = 20) = 0.87, *p* < 0.01), was stronger than for SUV_BW_ (r (df = 20) = 0.80, *p* < 0.01).

**Conclusion:**

Tumour lesion [^18^F]F-AraG uptake in patients with NSCLC is characterised by a 2T3k model. TBR and TPR show most potential for simplified quantification of tumour lesion [^18^F]F-AraG uptake in patients with NSCLC.

**Supplementary Information:**

The online version contains supplementary material available at 10.1007/s00259-024-06931-3.

## Introduction

Immune oncology (IO) therapy has become ever more important in the treatment of patients with lung cancer and other tumour types, and its use increases rapidly. However, while many patients benefit from IO treatment, a large group of patients does not [1, 2]. Insights into changes in the tumour micro-environment (TME) and lymphoid tissues triggered by an IO treatment will improve our understanding on the efficacy of IO within the lesions, and may guide patient selection for IO therapy.

The current standard to investigate the TME is by immunological assessment of biopsied material. While this method is useful for initial tumour typing and mutation analysis, it is not suited for assessing the TME. The TME is a complex heterogeneous structure that can vary in its composition within and between tumour lesions. Thus, a small tissue sample may not be representative for the biopsied tumour lesion nor for other tumour lesions in the body [3, 4]. Moreover, a biopsy is an invasive procedure and success depends on the location of the tumour. As such, biopsies are performed for one accessible lesion only and are not repeated more than necessary.

Positron emission tomography (PET) enables assessment of molecular processes in vivo, and does not suffer from these drawbacks. Various PET tracers have been explored for their ability to visualize TME processes, and to study IO treatment effects [5]. A new PET tracer, 2’-deoxy-2’-[^18^F]fluoro-9-β-D-Arabinofuranosyl-guanine ([^18^F]F-AraG), an analogue of AraG, the active substance of the drug Nelarabine, has been developed to image activated T-cells [6, 7]. Nelarabine is used in the treatment of T-cell acute lymphoblastic leukaemia and T-cell lymphoblastic lymphoma [8]. [^18^F]F-AraG enters the T-cell via nucleoside transporters. Inside the cell, it is phosphorylated primarily by mitochondrial deoxyguanosine kinase (dGK), or by cytosolic deoxycytidine kinase (dCK), and trapped intracellularly. The highest accumulation was found in activated CD8^+^ T-cells [7, 9]. Administration of tracer amounts of [^18^F]F-AraG and subsequent PET imaging allows non-invasive tracking of these activated T-cells, without having impact on the function and survival of the cells [9].

CD8^+^ T-cells, key components in anti-cancer immunity, are responsible for the destruction of cancer cells. For patients with non-small cell lung cancer (NSCLC), a baseline level of immune activation is expected around the tumour and in lymphoid organs, such as the spleen, bone marrow and lymph nodes. Patients responding well to IO therapy are likely to show higher levels of activated CD8^+^ T-cells in the TME [10]. Measuring the uptake of [^18^F]F-AraG in tumours could provide valuable insights into the presence and levels of activated T-cells as well as on the changes triggered by IO therapy. For quantification of PET tracer uptake, pharmacokinetic modelling is considered the most accurate quantification method [11]. However, this approach is complex and demanding for the patients, since it requires dynamic PET imaging and high-frequent blood sampling. Consequently, the use of simplified uptake measures, such as the standardised uptake value (SUV) or tumour-to-blood ratio (TBR), is desirable. Before these measures can be applied, they should be validated against findings from pharmacokinetic modelling.

In this study, we aimed to determine the optimal pharmacokinetic model to quantify tumour lesion [^18^F]F-AraG uptake in patients with NSCLC, and to validate simplified uptake measures at different time intervals against the dynamic pharmacokinetic uptake parameter.

## Methods

### Clinical trials

Thirteen patients with NSCLC from two clinical trials carried out at the Amsterdam University Medical Center, were included in the present study. The clinical trials were approved by the ethical review board of the Amsterdam University Medical Center and informed consent was obtained from all patients. The ATTAIN trial was registered under clinicaltrials.gov number NCT05157659. The SHARP trial was registered under clinicaltrials.gov number NCT05701176. The details of both trials are described in supplemental material SM1.

### Imaging protocols

Ten patients with early stage NSCLC with a resectable tumour were enrolled in the ATTAIN trial. PET imaging was acquired using an Ingenuity TF PET/CT scanner (Philips Healthcare). The axial field of view (FOV) was positioned over the thorax containing the primary tumour. A low dose CT (ldCT) scan was obtained for attenuation correction followed by a dynamic [^18^F]F-AraG PET scan. For the first five patients, the duration of the dynamic PET scan was 90 min. Initial analyses showed that the scan time could be reduced, hence, for all other patients, the scan duration was 70 min. One patient did not complete the full 70 min and was scanned 60 min. Six patients underwent a second scan within four days after the first dynamic PET scan. During scanning, blood samples were manually drawn at 5, 10, 15, 25, 40, 60 and 90 min post injection (p.i.). For seven patients, both arterial and venous sampling was obtained for correlation purposes. From the other three patients, the full sampling protocol could not be performed, so either arterial or venous sampling was collected.

Three patients with advanced stage NSCLC enrolled in the SHARP trial. PET imaging was acquired using a 106 cm long axial FOV Biograph Vision Quadra PET/CT scanner (Siemens Healthineers). A ldCT scan was obtained for attenuation correction followed by a 70 min dynamic [^18^F]F-AraG PET scan. During scanning, venous blood samples were manually drawn at 5, 10, 15, 25, 40 and 60 min p.i.

In both trials, PET scans were corrected for detector normalization, decay, dead time, attenuation, randoms and scatter. PET scans from the Ingenuity TF PET/CT were reconstructed using 3D-RAMLA and had a voxel size of 4 × 4 × 4 mm^3^ with a spatial resolution of 4.6–5.7 mm in full width at half maximum (FWHM). PET scans from the Biograph Vision Quadra PET/CT were reconstructed using PSF-TOF + 4 mm Gauss (EARL2 [12, 13]) and had a voxel size of 3.3 × 3.3 × 2 mm^3^ with a spatial resolution of 3.3–4.6 mm in FWHM. Depending on the respective scan times, all scans were reconstructed into 19, 20 or 22 frames (1 × 15, 3 × 5, 3 × 10, 4 × 60, 2 × 150, 2 × 300, and 4, 5 or 7 × 600 s).

### Blood sampling

For each sample, radioactivity concentrations (AC) in plasma and whole-blood were determined using a γ-counter. Furthermore, plasma was analysed for radiolabelled metabolites of [^18^F]F-AraG by solid-phase extraction (SPE). In brief, 1 mL of plasma was diluted with 2 mL water and loaded onto an activated Sep-Pak tC18 cartridge (Waters, Milford, MA, USA, 6 cc, 1 g). Vacuum was applied and the diluted plasma was passed over the SPE column, whereafter the cartridge was washed with 5 mL of water. The column was subsequently eluted with 5 mL methanol. Radioactivity in all three fractions (plasma, water, methanol) was measured. The first two fractions represent polar radiolabelled metabolites of [^18^F]F-AraG and the third intact [^18^F]F-AraG.

### Region delineation

[^18^F]F-AraG PET scans were visually assessed by a nuclear medicine physician for uptake in the primary tumour, metastases, lymph nodes and healthy organs. The corresponding volumes of interest (VOI) were manually delineated on the dynamic [^18^F]F-AraG PET, using the ACCURATE tool [14]. Both anatomical information from the ldCT scan and uptake on the PET scans were used to locate the VOI. In case of a small mismatch between ldCT and PET because of breathing motion, delineations were corrected by delineating only the overlap between ldCT and PET. Mean lesion AC over time (time-activity curves (TACs)) were obtained for each VOI for the duration of the dynamic PET scan.

A region of interest with a diameter of 17 to 20 mm was placed within the lumen of the ascending aorta on 5 to 8 consecutive axial slices (volumes ranging from 4.2 to 6.7 mL) to derive the blood AC from the images. These data will be used to generate an image derived input function (IDIFs).

Organs of interest within the FOV were manually delineated on the dynamic [^18^F]F-AraG PET scan using the ACCURATE tool [14]. Delineations of the lungs were obtained using a standard ldCT scan threshold. The liver, spleen, kidneys and muscle tissue (i.e., the biceps and triceps of both arms) were manually delineated based on the anatomical location on the ldCT scans, for the areas of the organs that were within the FOV. The delineations were corrected based on uptake on the [^18^F]F-AraG PET scan. The myocardium and thyroid glands were delineated based on uptake on the [^18^F]F-AraG PET scan. The bone marrow was delineated by placing 3 fixed size VOIs within in 3 separate thoracic vertebrae. SUV_BW_ was obtained for each organ for the duration of the dynamic PET scan to evaluate the biodistribution of [^18^F]F-AraG. All delineations were adjusted to minimize spill-over from adjacent blood vessels using the first timeframes (40–60 s p.i.) of the dynamic scan.

### Input function for modelling

All venous samples were corrected for the bias between venous and arterial sampling (see supplemental Fig. S1 and S2) by applying a correction factor to the whole-blood and plasma samples for each time point. For the ATTAIN trial, acquired using the Ingenuity TF PET/CT scanner, quantification of the IDIF showed misalignment in the shape of the curve when compared to whole-blood samples. This appeared to result from low signal-to-noise ratios on the PET at later time points due to scatter correction issues as assessed with phantom experiments (data not shown). Consequently, high noise levels were present in the aorta due to surrounding high-uptake areas (i.e., the myocardium and liver), which only influenced the IDIF at later time points with low AC in the ascending aorta (~ 700 Bq/mL). To correct for this, a bi-exponential fit through the whole-blood samples (from 5 min p.i. until the end of the scan) was combined with the first five minutes of the IDIF (not influenced by scatter correction issues) to obtain a whole-blood input functions. For the SHARP trial, acquired using the Biograph Vision Quadra PET/CT scanner, this issue did not appear. A single exponential function fitted to the samples was compared to the IDIF-tail (> 800s) to obtain a calibration factor for the IDIF. This calibration factor was derived by minimizing the difference in IDIF and sample activity concentration values. The IDIFs were scaled with this calibration factor (towards the samples) to obtain a whole-blood input function. Subsequently, all whole-blood input functions were corrected for plasma/whole-blood ratios, which were extrapolated to all time points, and parent fractions, through which a Hill function was fitted. For two patients (i.e. ATT03 and ATT04), assessment of the parent fraction was unsuccessful due to low extraction efficiency of the SPE material. The method was optimised for the following patients. For these two patients, the median parent fraction of the other patients was used for correcting the input functions. Metabolite corrected plasma input functions were used for pharmacokinetic modelling.

### Pharmacokinetic modelling

Pharmacokinetic modelling was performed on the lesion TACs using in-house developed software build in MATLAB (MATLAB version 7.04, The MathWorks, Inc., Natick, Massachusetts, United States). The tool applies weighted non-linear regression with constraints on the pharmacokinetic rate constants [15]. A single-tissue reversible model (1T2k), a two-tissue irreversible model (2T3k) and a two-tissue reversible model (2T4k) were fitted to the TACs with an additional fit parameter for the blood volume fraction (V_b_) to assess the preferred pharmacokinetic model [11]. The following pharmacokinetic rate constants were derived from the analyses; *K*_*1*_ [min^− 1^] representing the [^18^F]F-AraG influx from plasma to tissue, *k*_*2*_ [min^− 1^] representing the [^18^F]F-AraG efflux from tissue to plasma, *k*_*3*_ [min^− 1^] representing the rate [^18^F]F-AraG uptake to activated T-cells and *k*_*4*_ [min^− 1^] representing the release rate of [^18^F]F-AraG from activated T-cells. The rate constant *K*_*i*_, representing the net influx rate, and the *V*_*T*_, representing the distribution volume, were calculated from the obtained pharmacokinetic rate constants [11]:

$$\:{K}_{i}=\frac{{K}_{1}*{k}_{3}}{{k}_{2}+{k}_{3}}\quad\quad{V}_{T}=\frac{{K}_{1}}{{k}_{2}}*(1+\frac{{k}_{3}}{{k}_{4}})$$

### Simplified uptake measures

The SUVs corrected for body weight (SUV_BW_), body surface area (SUV_BSA_), and lean body mass (SUV_LBM_) were derived for the following time intervals: 20–30, 30–40, 40–50, 50–60 and 60–70 min p.i. Tumour-to-blood ratio (TBR) and tumour-to-plasma ratio (TPR) were derived for the following time intervals: 20–30, 40–50 and 60–70 min p.i.

### Statistical analyses

The preferred kinetic model was selected based on the goodness of fit, both visually assessed and tested with the Akaike information criterion (AIC) [16]. Since some tumour lesions were included for both test and re-test, and arterial and venous sampling, only one data point per lesion was included for correlation analyses. If available, the scan corresponding with the arterial sampling was selected. Pearson correlation was performed to assess the association between simplified uptake measures and the outcome parameter of the optimal pharmacokinetic model. The significance level (p-value) was set at 0.05. Statistical analyses were performed using R Statistical Software (v3.6.1; R Core Team, 2019).

## Results

### Patient characteristics

**Fig. 1:**
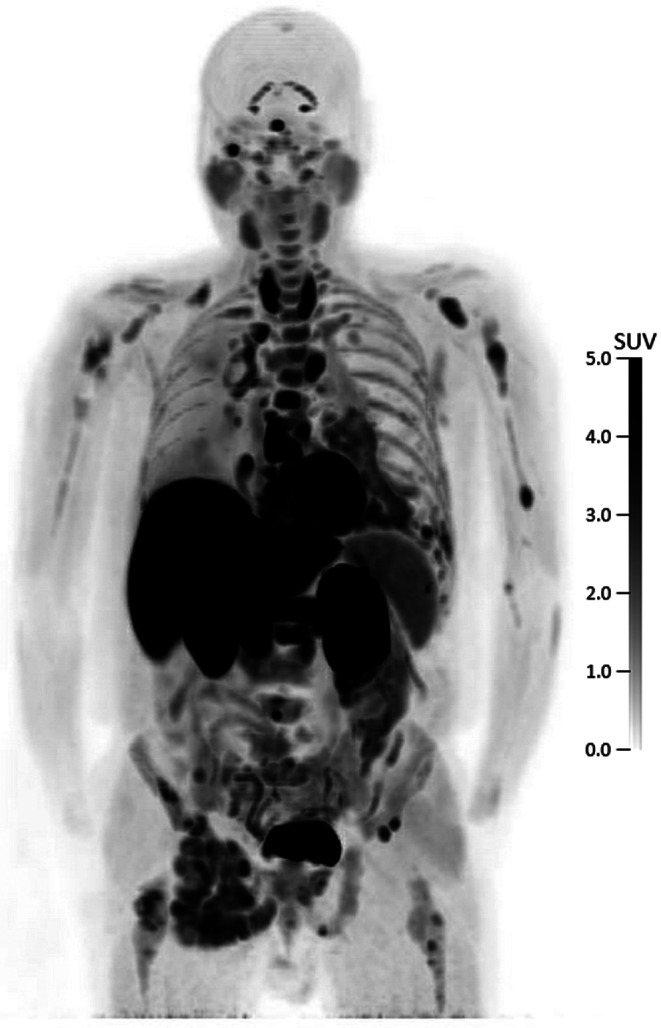
Maximum intensity projection of a 60–70 min p.i. [^18^F]F-AraG PET scan of a 63 yrs old male patient with advanced stage NSCLC. Physiological uptake can be observed in the liver, kidneys, spleen, bladder, myocardium, bone marrow and thyroid, pituitary and salivary glands. Additionally, high uptake can be seen in tumour lesions (lung) and bone metastases (right jaw, both humeri, sternum, multiple vertebrae and right acetabulum)

An overview of the patient characteristics and scanning procedures is shown in Table 1, and Fig. 1 shows an example [^18^F]F-AraG PET scan. For all but one patient in the ATTAIN trial, only the primary tumour was identified within the FOV. For patient ATT09, the primary tumour could not be quantified due to pleural uptake around the tumour, but one malignant lymph node was identified and quantified instead. The primary tumour of patient ATT01 was located at the edge of the FOV and near the liver, resulting in spill-over from the liver. The tumour could therefore not be quantified and was excluded from further analyses. Two lung lesions, highly suspected for NSCLC metastases, were identified from patient SHARP01; one lung lesion and six lymph nodes, all suspected for malignancy, were identified from patient SHARP02; and one lung lesion and four lymph nodes, suspected for malignancy, were identified from patient SHARP03. The mean injected [^18^F]F-AraG activity was 184.3 MBq (standard deviation (SD) = 12.1), with mean molar activity of 67.0 GBq/µmole (SD = 29.9) and mean tracer mass dose of 1.15 µg (SD = 0.66).

**Table 1:** Patient characteristics and overview of acquired scans

Patient	Age	Sex	Delineated lesion	Scan 1	Scan 2	Duration PET scan	Pre-treatment
ATT01*	75	M	NA	Arterial	Venous	90 min	RT + IO
ATT03^#^	63	M	T	Arterial + venous	Venous	90 min	None
ATT04^#^	72	F	T	Venous	Arterial + venous	90 min	None
ATT05	66	F	T	Venous	Arterial + venous	90 min	None
ATT06	71	M	T	Arterial	-	90 min	None
ATT07	69	F	T	Venous	-	60 min	None
ATT09	76	M	N	Arterial + venous	-	70 min	None
ATT10	76	M	T	Venous	Arterial + venous	70 min	RT + IO
ATT11	61	F	T	Arterial + venous	-	70 min	RT + IO
ATT12	77	M	T	Venous	Arterial + venous	70 min	None
SHARP01	63	M	2x M	Venous	-	70 min	None
SHARP02	84	M	M, 6x N	Venous	-	70 min	None
SHARP03	78	M	M, 4x N	Venous	-	70 min	None

T = primary lung tumour, N = (malignant) lymph node, M = (distant) metastasis, NA = not available, RT = radiotherapy, IO = immunotherapy. * Data was excluded because of unquantifiable tumour uptake. ^#^ Median parent fractions of the other patients were used for correcting the input functions to perform pharmacokinetic modelling

### Blood sample analysis

**Fig. 2:**
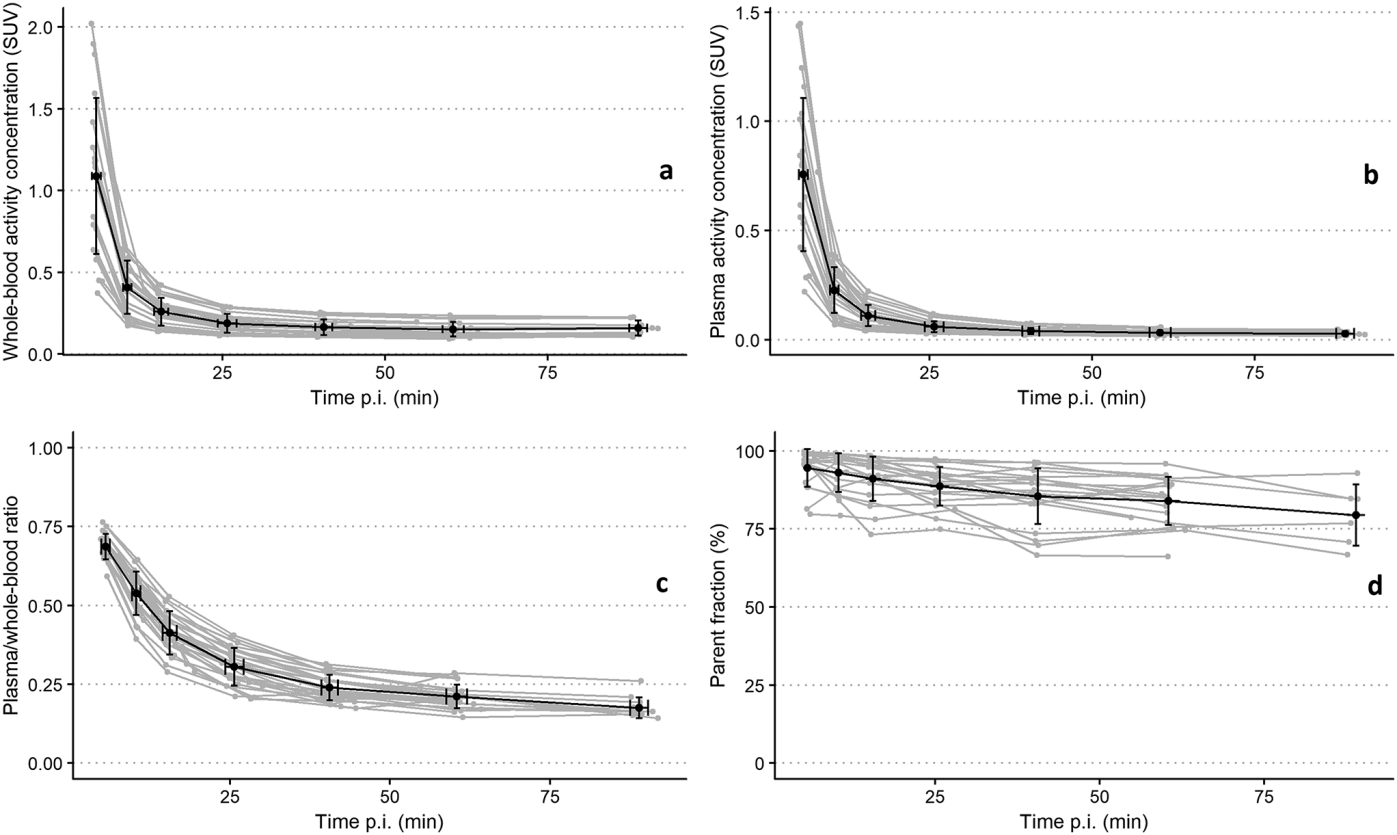
Blood sampling time activity curves. Mean [^18^F]F-AraG activity concentration in whole-blood (**a**) and plasma (**b**) expressed in SUV_BW_, plasma/whole-blood ratios (**c**) and [^18^F]F-AraG parent fractions (**d**) over time. Data of thirteen patients are shown, except for (**d**) where data was missing for patients ATT03 and ATT04. The error bars represent one SD from the mean. Individual patient data is represented by grey lines

Measured whole-blood and plasma AC showed rapid clearance of [^18^F]F-AraG, decreasing from a mean SUV_BW_ of 1.09 (SD = 0.48) at 5 min p.i. to a mean SUV_BW_ of 0.19 (SD = 0.06) at 25 min p.i. (Figure 2a and b). The mean ratio between plasma and whole-blood AC decreased from 0.69 (SD = 0.04) to 0.18 (SD = 0.03) during the course of the scan (Fig. 2c). Parent fractions remained relatively high and constant over time, showing a mean decrease from 94% (SD = 6.1%) to 79% (SD = 9.8%) (Fig. 2d). Arterial and venous blood AC as well as parent fractions obtained from six patients showed strong correlations (supplemental Fig. S1). For pharmacokinetic modelling, input functions were corrected for the bias between venous and arterial sampling (see supplemental Fig. S2).

### Biodistribution

**Fig. 3:**
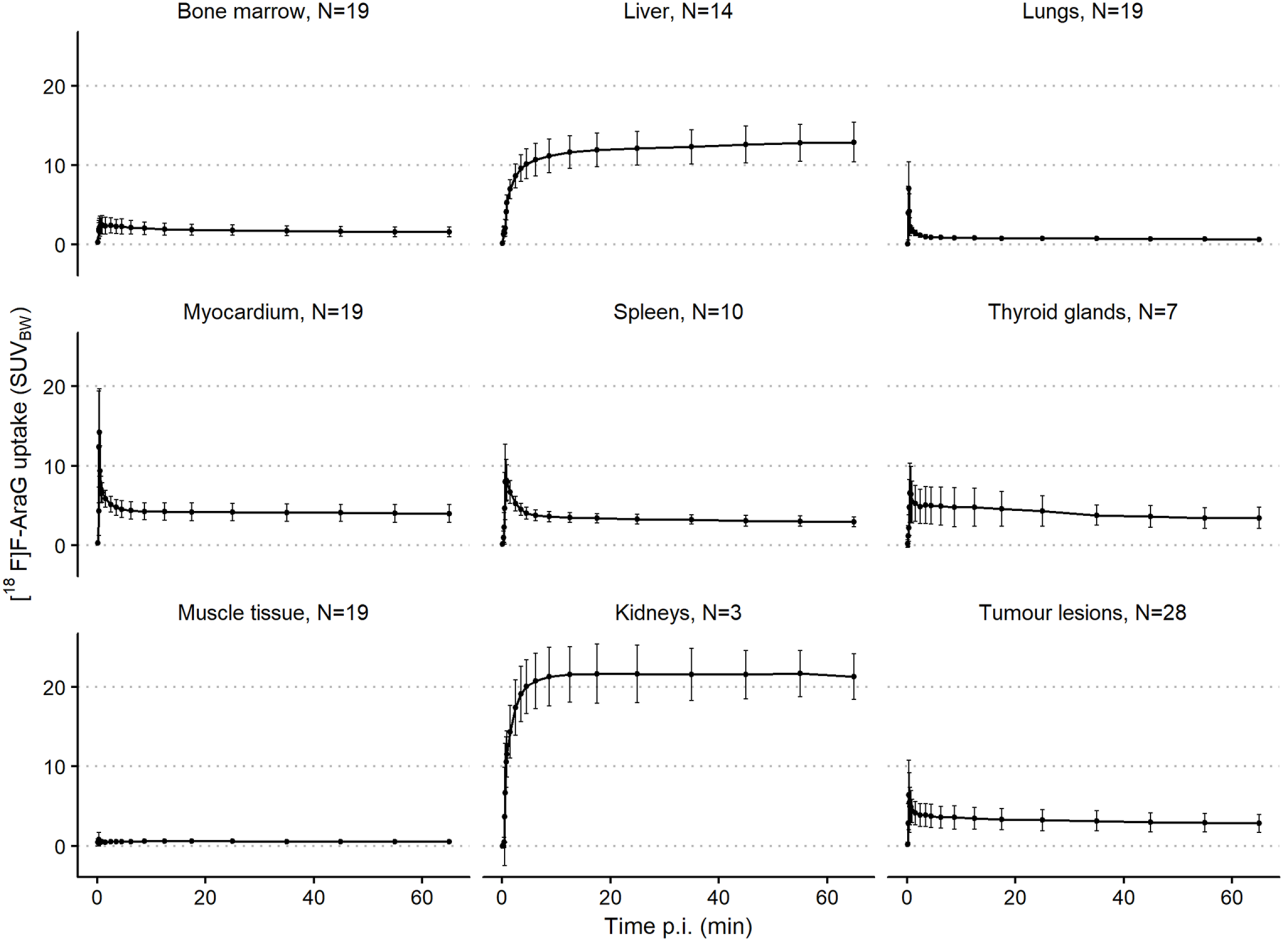
Mean organ and tumour [^18^F]F-AraG uptake over time expressed in SUV_BW_ derived from N scans. The error bars represent one standard deviation from the mean

For the ATTAIN trial, lungs, myocardium, bone marrow and muscle tissue were delineated in all scans. Delineation of the other organs depended on the placement of the FOV. The number of scans on which each organ of interest was delineated, is indicated by N in Fig. 3. For the three patients included from the SHARP trial, eight organs of interest were delineated.

Mean uptake in the liver showed an slight increase over time, from a SUV_BW_ of 11.9 at 17.5 min p.i. to a SUV_BW_ of 12.9 at 65 min p.i. (see Fig. 3). [^18^F]F-AraG uptake in all other organs remained constant or decreased during the scan. Uptake in liver and kidneys exceeded the range of uptake in tumour lesions. Uptake in lungs and muscle tissue was lower than the range of tumour lesion uptake, and uptake in bone marrow, spleen, thyroid glands and myocardium were within the range of tumour lesion uptake.

### Pharmacokinetic modelling

**Fig. 4:**
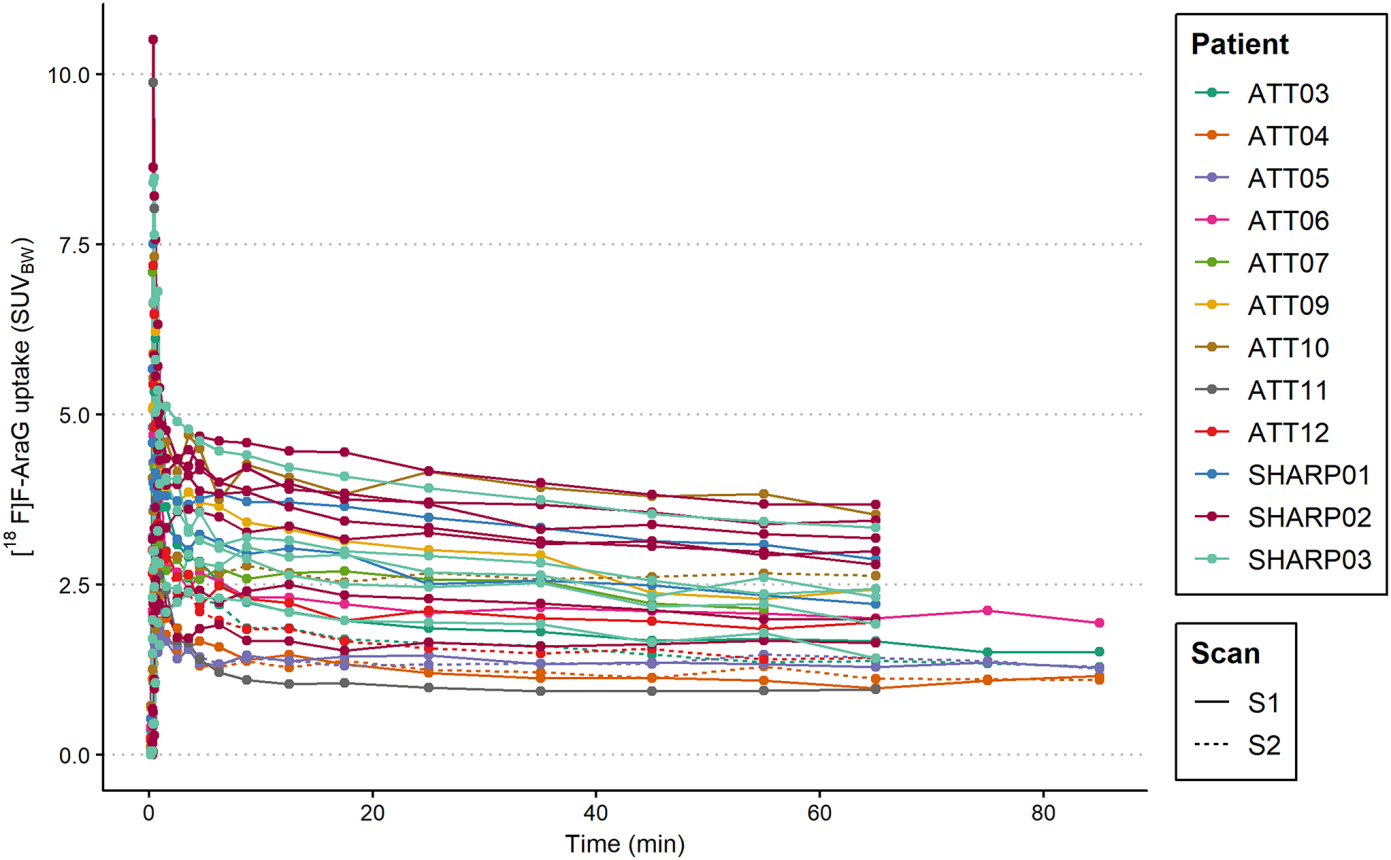
[^18^F]F-AraG uptake in tumour lesions over time expressed in SUV_BW_

The tumour lesion TACs are shown in Fig. 4. Pharmacokinetic modelling provided good fits for all TACs to the 2T3k-model and 2T4k-model based on AIC results and visual assessment. Based on the AIC, the 2T3k-model was the preferred model for most of the TACs (24 out of 35), the 2T4k-model was preferred for 10 out of 35 TACs, and the 1T2k-model was preferred for one of the TACs (see supplemental Table S1). The 2T3k-derived K_i_ ranged from 0.02 to 0.15 h^− 1^, while values for 2T4k-derived V_T_ were less reliable as they showed a large range from 2.77 to 1.30*10^3^ (see supplemental Table S2). Additionally, the added rate constant responsible for the reversible kinetics in the 2T4k-model compared to the 2T3k-model, the k_4_, was small (mean = 0.02, SD = 0.03), supporting an irreversible uptake behaviour in tumour lesions. For four patients who underwent a 90-minute dynamic scan, a very strong positive correlation was found between 2T3k-derived *K*_*i*_ obtained from 0 to 70 min versus 0 to 90 min p.i. (see supplemental Fig. S3).

### Comparison between pharmacokinetic modelling and simplified uptake measures

**Fig. 5:**
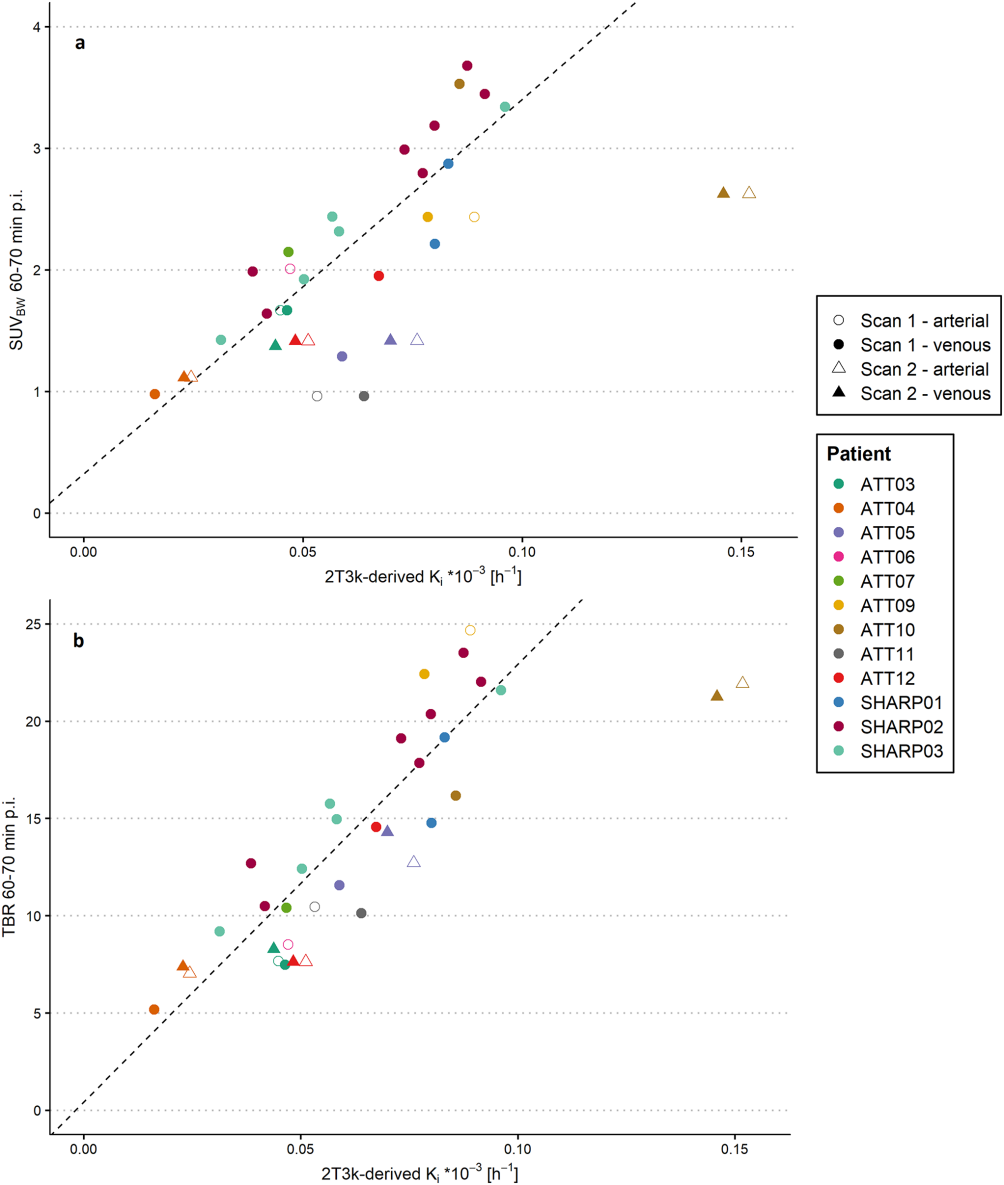
Correlation between simplified uptake measures and 2T3k-derived *K*_*i*_. Scatter plots showing tumour lesion [^18^F]F-AraG uptake (**a**) expressed in SUV_BW_ at 60–70 min p.i. against 2T3k-derived *K*_*i*,_ and (**b**) expressed in TBR at 60–70 min p.i. against 2T3k-derived *K*_*i*_. *Note* that multiple data points from the same lesion are represented in the figure, but only one data point per lesion was used for correlation analyses. Multiple tumour lesions were quantified for the SHARP patients and are presented in the same colour. For patient ATT07, the presented SUV_BW_ and TBR were obtained at 50–60 min p.i

The simplified uptake measures (SUV_BW_, SUV_BSA_, SUV_LBM_, TBR and TPR) were calculated for different time intervals and compared to the 2T3k-derived *K*_*i*_. Scatter plots of SUV_BW_ and TBR from 60 to 70 min p.i. versus 2T3k-derived *K*_*i*_ are shown in Fig. 5a and b, respectively. There was a strong correlation between SUV_BW_ 60–70 min p.i. and 2T3K-derived *K*_*i*_ (r (df = 20) = 0.80, *p* < 0.01), and between TBR 60–70 min p.i. and 2T3K-derived *K*_*i*_ (r (df = 20) = 0.87, *p* < 0.01). All Pearson correlations coefficients for comparisons between each simplified uptake measure and the 2T3K-derived *K*_*i*_ are presented in supplemental Table S3. For SUV_BW_, SUV_BSA_ and SUV_LBM_, correlation coefficients and slopes were similar for different uptake intervals. For TBR and TPR, the slope increased at later time intervals, but correlation coefficients were similar.

### Preliminary test-retest analyses

Preliminary results from test and retest scans showed relatively consistent correlations between simplified measures and the 2T3k-derived *K*_*i*_, except for patient ATT10 (supplemental Fig. S4). Test-retest results for the simplified uptake measures showed relative differences in SUV_BW_ from 10.2 to 27.4%, and from 23.7 to 47.6% for TBR (see supplemental Fig. S5 and supplemental Table S4).

## Discussion

The aim of this study was to identify the optimal pharmacokinetic model to describe [^18^F]F-AraG uptake in tumour lesions and to validate simplified uptake measures. Tumour lesion uptake of [^18^F]F-AraG in patients with NSCLC was best characterised by a two-tissue irreversible (2T3k) model for the majority of the tumour TACs. All simplified measures at different time intervals between 20 and 70 min p.i. showed strong correlations with the 2T3k-derived *K*_*i*_. TBR or TPR showed most potential for reliably quantifying [^18^F]F-AraG uptake in tumour lesions.

[^18^F]F-AraG AC in blood showed a rapid decrease in the first 20 min p.i. and a slower decrease thereafter, which is in line with the data reported previously in 6 healthy volunteers [7]. Venous and arterial data showed strong correlations close to the line of identity, therefore, the arterial sampling can be replaced with less invasive venous sampling. On average, plasma/whole-blood ratios were 0.69 at 5 min p.i. and decreased to 0.18 at 70 min p.i. This occurred very quickly, leading to a decreased availability of free [^18^F]F-AraG for the lesions. This decrease may have resulted from uptake of [^18^F]F-AraG into activated T-cells inside the blood. Unfortunately, no measurements of the presence of T-cells in the blood were available yet. Metabolite analysis of collected blood samples revealed a low rate of metabolism (a mean parent fraction of 79% at 70 min p.i.), so most of the [^18^F]F-AraG available in blood remained intact.

[^18^F]F-AraG uptake in organs was relatively constant from 20 min p.i. onwards. Uptake in liver, lungs, spleen, kidneys, myocardium, muscle tissue and thyroid glands was similar to what was measured in healthy controls (7, 19). However, Levi et al. reported lower uptake in bone marrow as compared to our study [7]. This difference may be due to higher baseline immune activation status within our patient population due to their disease. The uptake in lung tissue and muscle tissue was lower than in the tumour lesions, indicating that malignant lesions within these tissues can be discriminated from background tissue. The highest [^18^F]F-AraG uptake was found in the liver in accordance with previous observations [17, 18], which was, on average, three times higher than the uptake in tumour lesions. Therefore, the quantification of one tumour lesion located close to the liver suffered from spill-over effects from the liver and was excluded from the analysis.

Our findings are consistent with the biological hypothesis that [^18^F]F-AraG is transported into activated T-cells and subsequently phosphorylated by dGK, resulting in its accumulation within the cell [9]. The activation state of dGK is associated with mitochondrial biogenesis that is tightly controlled by the sterile alpha motif and HD-domain containing protein (SAMHD1) [19]. SAMHD1 was recently identified to play a critical role in [^18^F]F-AraG uptake [7]. SAMHD1 can dephosphorylate [^18^F]F-Ara-GTP into [^18^F]F-AraG, enabling its release from the cell resulting in a reversible uptake behaviour. However, activated T-cells have an increased need in mitochondrial biogenesis and activity and, therefore, downregulate SAMHD1 as shown for stimulated CD8^+^ T-cells [7]. The observed tumour lesion [^18^F]F-AraG uptake in the present study was best characterised by a two-tissue irreversible model. This is consistent with the biological processes occurring in activated T-cells. On the other hand, Omidvari et al. reported a two-tissue reversible model for [^18^F]F-AraG in the tumour of one patient with NSCLC. We cannot exclude that differences in the results could be attributed to differences in the input functions used for the kinetic modelling. While Omidvari et al. applied an IDIF [18], our study scaled the IDIF to blood samples and included corrections for the decreases in plasma/whole-blood ratio and parent fraction. These decreases cannot be accounted for when only using an IDIF, which potentially affects the kinetic modelling. To substantiate this, our pharmacokinetic modelling results without applying corrections for parent fraction and plasma/whole-blood ratio showed a preference for the 2T4k model in the majority of the TACs (see supplemental Table S5).

Pharmacokinetic modelling provides comprehensive insights into tracer kinetics and uptake behaviour. However, in daily practice, a simplified approach is more practicable. Hence, we evaluated the association of simplified uptake measures with the 2T3k-derived *K*_*i*_. All simplified measures correlated strongly with the 2T3k-derived *K*_*i*_, suggesting their potential for reliably quantifying tumour lesion [^18^F]F-AraG uptake. SUV usage is advantageous, because it can be obtained from a single PET scan and is a commonly used metric for quantifying PET uptake. Notably, there were no differences found between the different normalization methods (i.e. BW, LBM or BSA) for the SUV. However, the lower uptake in adipose tissue (supplemental Fig. S6) suggests that normalization using LBM or BSA might be more appropriate, particularly in patient cohorts exhibiting greater variability in body weight. However, when comparing SUV to 2T3k-derived *K*_*i*_, some variability could be noted along the regression line. This variability decreased and the correlation improved when using a TBR or TPR, as it accounted for differences in blood clearance. Suggesting that TBR or TPR are even more suitable measures for quantifying [^18^F]F-AraG uptake in tumour lesions.

Although earlier post-injection time intervals can be used for reliably quantifying tumour lesion [^18^F]F-AraG uptake, we recommend the 60–70 min p.i. interval, as this aligns conveniently with standard scanning protocols, such as the commonly used 60 min p.i. interval in [^18^F]FDG-PET imaging [20] and as already used in [^18^F]F-AraG-PET imaging [17]. Furthermore, the slopes of the correlations for different time intervals were similar, indicating that the tumour lesion [^18^F]F-AraG uptake may be compared between different uptake times. This flexibility allows for quantifying [^18^F]F-AraG uptake even if the timing of the PET scan varies.

A number of limitations of the current study can be considered regarding the heterogeneity of the dataset. Firstly, a test-retest analysis was performed for only four patients, showing moderate reliability for the SUV_BW_ and TBR at 60–70 min p.i. Yet, the test-retest of the kinetic modelling demonstrated a consistent relationship between simplified uptake measures and the 2T3k-derived *K*_*i*_. Future studies should investigate the test-retest reliability in a larger cohort. Secondly, this study included patients from two different clinical trials, which involved the use of PET scanners with varying scan performances. Since no established harmonisation program for dynamic PET scans exists, we applied for both scanners the reconstruction protocol that provided the best images (as a trade-off between contrast recovery and noise). The reconstruction for the Philips Ingenuity scans were compatible with EARL1 standards, while the Biograph Vision Quadra scans were compatible with EARL2 standards. As the validation of simplified uptake measures is based on the same reconstructions per patient, it can be expected that SUV, TBR and *K*_*i*_ are similarly affected by the differences in reconstructed resolution and that the impact on their association is minimal. Indeed, additional analyses confirmed that the relationship between simplified uptake measures and 2T3k-derived *K*_*i*_ was consistent for a EARL1-compatible reconstruction of the Biograph Vision Quadra scans, compared to the original EARL2-compatible reconstructed scans (see supplemental Fig. S7). This indicates that the lack of harmonization between scanners does not impact our conclusions. Thirdly, the *k*_*3*_ represents the rate of uptake from the tumour tissue into activated T-cells, while the *K*_*i*_ is the net rate of uptake from plasma into activated T-cells and is therefore dependent on the perfusion of the tumour. Even though the *k*_*3*_ would better describe the actual uptake into activated T-cells, the *k*_*3*_ is much less precise and more difficult to obtain. On the other hand, the *K*_*i*_ is less affected by noise and can be obtained with higher precision. *K*_*i*_ or late uptakes are also perfusion dependent and, ideally, a perfusion measurement using e.g. ^15^O-water would be good to have to further validate *K*_*i*_. Additionally, the *K*_*i*_ can also be derived from linearized approaches such as Patlak graphical analysis [21], which is less influenced by noise and can be calculated from parametric PET imaging. Lastly, although there is also a difference in tumour staging between the two trials, we do not expect this to impact our study. While immune infiltration may vary between stages and lesions and lymph nodes, the kinetics of the tracer uptake into activated T-cells are unlikely to change. Nonetheless, the perfusion of the tumour may change due to the effect of treatment and this would affect the [^18^F]F-AraG uptake. Future research should therefore explore the impact of the T-cell activation status and whether the kinetics of [^18^F]F-AraG remain consistent under different conditions, such as during immunotherapy, and across various types of cancer.

## Conclusion

Tumour lesion [^18^F]F-AraG uptake in patients with NSCLC is characterised by a two-tissue irreversible model. All simplified measures, SUV corrected for BW, LBM or BSA, as well as the TBR and TPR, correlated strongly with the main uptake value of this model, *K*_*i*_. These correlations were consistent from 20 min post injection onwards. TBR and TPR showed the strongest correlations and thus have most potential for quantifying tumour lesion [^18^F]F-AraG uptake in patients with NSCLC.

## Electronic supplementary material

Below is the link to the electronic supplementary material.

Supplementary Material 1

## Data Availability

All data analysed during this study are available on reasonable request.

## References

[1] Brahmer J, Rodriguez-Abreu D, Robinson A, Hui R, Csőszi T, Fülöp A, et al. LBA51 KEYNOTE-024 5-year OS update: first-line (1L) pembrolizumab (pembro) vs platinum-based chemotherapy (chemo) in patients (pts) with metastatic NSCLC and PD-L1 tumour proportion score (TPS) ≥ 50%. Ann Oncol. 2020;31:S1181–2.

[2] Paz-Ares LG, Ramalingam SS, Ciuleanu T-E, Lee J-S, Urban L, Caro RB, et al. First-line nivolumab plus ipilimumab in advanced NSCLC: 4-year outcomes from the randomized, open-label, phase 3 CheckMate 227 part 1 trial. J Thorac Oncol. 2022;17:289–308. 34648948 10.1016/j.jtho.2021.09.010

[3] Anderson NM, Simon MC. The tumor microenvironment. Curr Biol. 2020;30:R921–5. 32810447 10.1016/j.cub.2020.06.081 PMC8194051

[4] Jiménez-Sánchez A, Memon D, Pourpe S, Veeraraghavan H, Li Y, Vargas HA, et al. Heterogeneous tumor-immune microenvironments among differentially growing metastases in an ovarian cancer patient. Cell. 2017;170:927–38. e20. 28841418 10.1016/j.cell.2017.07.025 PMC5589211

[5] Slebe M, Pouw JE, Hashemi SM, Menke-van der Houven CW, Yaqub MM, Bahce I. Current state and upcoming opportunities for immunoPET biomarkers in lung cancer. Lung Cancer. 2022;169:84–93. 35679715 10.1016/j.lungcan.2022.05.017

[6] Namavari M, Chang YF, Kusler B, Yaghoubi S, Mitchell BS, Gambhir SS. Synthesis of 2’-deoxy-2’-[18F]fluoro-9-beta-D-arabinofuranosylguanine: a novel agent for imaging T-cell activation with PET. Mol Imaging Biol. 2011;13:812–8. 10.1007/s11307-010-0414-x. 20838911 10.1007/s11307-010-0414-x

[7] Levi J, Duan H, Yaghoubi S, Packiasamy J, Huynh L, Lam T, et al. Biodistribution of a mitochondrial metabolic Tracer, [(18)F]F-AraG, in healthy volunteers. Mol Imaging. 2022;2022:3667417. 10.1155/2022/3667417. 36072652 10.1155/2022/3667417 PMC9400547

[8] Sanford M, Lyseng-Williamson KA, Nelarabine. Drugs. 2008;68(4). 10.2165/00003495-200868040-00004 18318562

[9] Levi J, Lam T, Goth SR, Yaghoubi S, Bates J, Ren G, et al. Imaging of Activated T Cells as an early predictor of Immune response to Anti-PD-1 therapy. Cancer Res. 2019;79:3455–65. 10.1158/0008-5472.CAN-19-0267. 31064845 10.1158/0008-5472.CAN-19-0267 PMC6606349

[10] Li F, Li C, Cai X, Xie Z, Zhou L, Cheng B et al. The association between CD8 + tumor-infiltrating lymphocytes and the clinical outcome of cancer immunotherapy: a systematic review and meta-analysis. EClinicalMedicine. 2021;41. 10.1016/j.eclinm.2021.101134 PMC8452798 34585125

[11] Gunn RN, Gunn SR, Cunningham VJ. Positron emission tomography compartmental models. J Cereb Blood Flow Metabolism. 2001;21:635–52. 10.1097/00004647-200106000-00002 11488533

[12] Boellaard R, Delgado-Bolton R, Oyen WJ, Giammarile F, Tatsch K, Eschner W, et al. FDG PET/CT: EANM procedure guidelines for tumour imaging: version 2.0. Eur J Nucl Med Mol Imaging. 2015;42:328–54. 25452219 10.1007/s00259-014-2961-x PMC4315529

[13] Kaalep A, Sera T, Rijnsdorp S, Yaqub M, Talsma A, Lodge MA, Boellaard R. Feasibility of state of the art PET/CT systems performance harmonisation. Eur J Nucl Med Mol Imaging. 2018;45:1344–61. 29500480 10.1007/s00259-018-3977-4 PMC5993859

[14] Boellaard R. Quantitative oncology molecular analysis suite: ACCURATE. Soc Nuclear Med; 2018.

[15] Yaqub M, Boellaard R, Kropholler MA, Lammertsma AA. Optimization algorithms and weighting factors for analysis of dynamic PET studies. Phys Med Biol. 2006;51:4217. 16912378 10.1088/0031-9155/51/17/007

[16] Akaike H. A new look at the statistical model identification. IEEE Trans Autom Control. 1974;19:716–23.

[17] Ronald JA, Kim BS, Gowrishankar G, Namavari M, Alam IS, D’Souza A, et al. A PET imaging strategy to visualize activated T cells in Acute Graft-versus-host Disease elicited by allogenic hematopoietic cell transplant. Cancer Res. 2017;77:2893–902. 10.1158/0008-5472.CAN-16-2953. 28572504 10.1158/0008-5472.CAN-16-2953 PMC5505323

[18] Omidvari N, Levi J, Abdelhafez YG, Wang Y, Nardo L, Daly ME et al. Total-body dynamic imaging and kinetic modeling of [18F] F-AraG in healthy individuals and a non–small cell Lung Cancer patient undergoing Anti–PD-1 immunotherapy. J Nucl Med. 2024;65(9):1481-1488. 10.2967/jnumed.123.267003 PMC11372257 39089813

[19] Rothenburger T, McLaughlin K-M, Herold T, Schneider C, Oellerich T, Rothweiler F, et al. SAMHD1 is a key regulator of the lineage-specific response of acute lymphoblastic leukaemias to nelarabine. Commun Biology. 2020;3:324. 10.1038/s42003-020-1052-8 PMC7314829 32581304

[20] Boellaard R. Standards for PET image acquisition and quantitative data analysis. J Nucl Med. 2009;50(Suppl 1):S11–20. 10.2967/jnumed.108.057182. 10.2967/jnumed.108.057182 19380405

[21] Patlak CS, Blasberg RG, Fenstermacher JD. Graphical evaluation of blood-to-brain transfer constants from multiple-time uptake data. J Cereb Blood Flow Metabolism. 1983;3:1–7. 10.1038/jcbfm.1983.1 6822610

