# Supplementary material for: Pharmacokinetic analysis and simplified uptake measures for tumour lesion [18F]F-AraG PET imaging in patients with non-small cell lung cancer
Source: Eur J Nucl Med Mol Imaging. 2024 Oct 8;52(2):719–29. doi: 10.1007/s00259-024-06931-3 (PMC11732896; doi:10.1007/s00259-024-06931-3)
Supplement: Supplementary file 1 — Supplementary Material 1 [file 259_2024_6931_MOESM1_ESM.docx]

**SM1: Clinical trials**

*ATTAIN*

The ATTAIN trial, registered under EUDRACT number 2021-001489-40 and CCMO number NL77310.029.21, known under the full title: “A clinical imaging study using [^18^F]F-AraG PET to visualize Tumor infiltrating T-cell Activation in Non-small cell lung cancer”, is a single arm imaging study where 10 resectable early-stage NSCLC patients receive 2 dynamic [^18^F]F-AraG PET scans prior to their routine tumour resection. The main objective of this trial is to perform full kinetic modelling on [^18^F]F-AraG uptake in tumor lesions, to assess the test-retest variability of the scans and to correlate the relation between [^18^F]F-AraG uptake and number of CD8^+^ T-cells and other immune cells measured by immunohistochemistry and gene expression. Primary inclusion criteria is a histologically confirmed NSCLC, ≥ 2 cm, scheduled for resection. Primary exclusion criteria are the use of immunosuppressive medications within 14 days of screening, pregnancy and psychiatric or substance disorders that would interfere with cooperation with the requirements of the trial.

*SHARP*

The SHARP trial, registered under EUDRACT number 2021-003986-36 and CCMO number NL78588.029.21, known under the full title: “A clinical imaging Study of the changes in [^18^F]F-AraG uptake following anti-PD-1 therapy in Non-small cell lung cancer”, is a single arm imaging study where 15 advanced stage NSCLC patients, scheduled to receive anti-PD-1 therapy, receive 3 dynamic [^18^F]F-AraG PET scans at baseline, at 2 weeks and at 6 weeks after the start of their treatment. The main objective of this trial is to assess relative change in [^18^F]F-AraG uptake in tumor lesions upon anti-PD-1 therapy and to assess the relation between baseline [^18^F]F-AraG uptake, change in uptake and response to anti-PD-1 therapy. Primary inclusion criteria is a histologically confirmed stage IIIB-IV NSCLC, planned to receive anti-PD-1 monotherapy. Primary exclusion criteria are the use of immunosuppressive medications within 14 days of screening, pregnancy and psychiatric or substance disorders that would interfere with cooperation with the requirements of the trial.


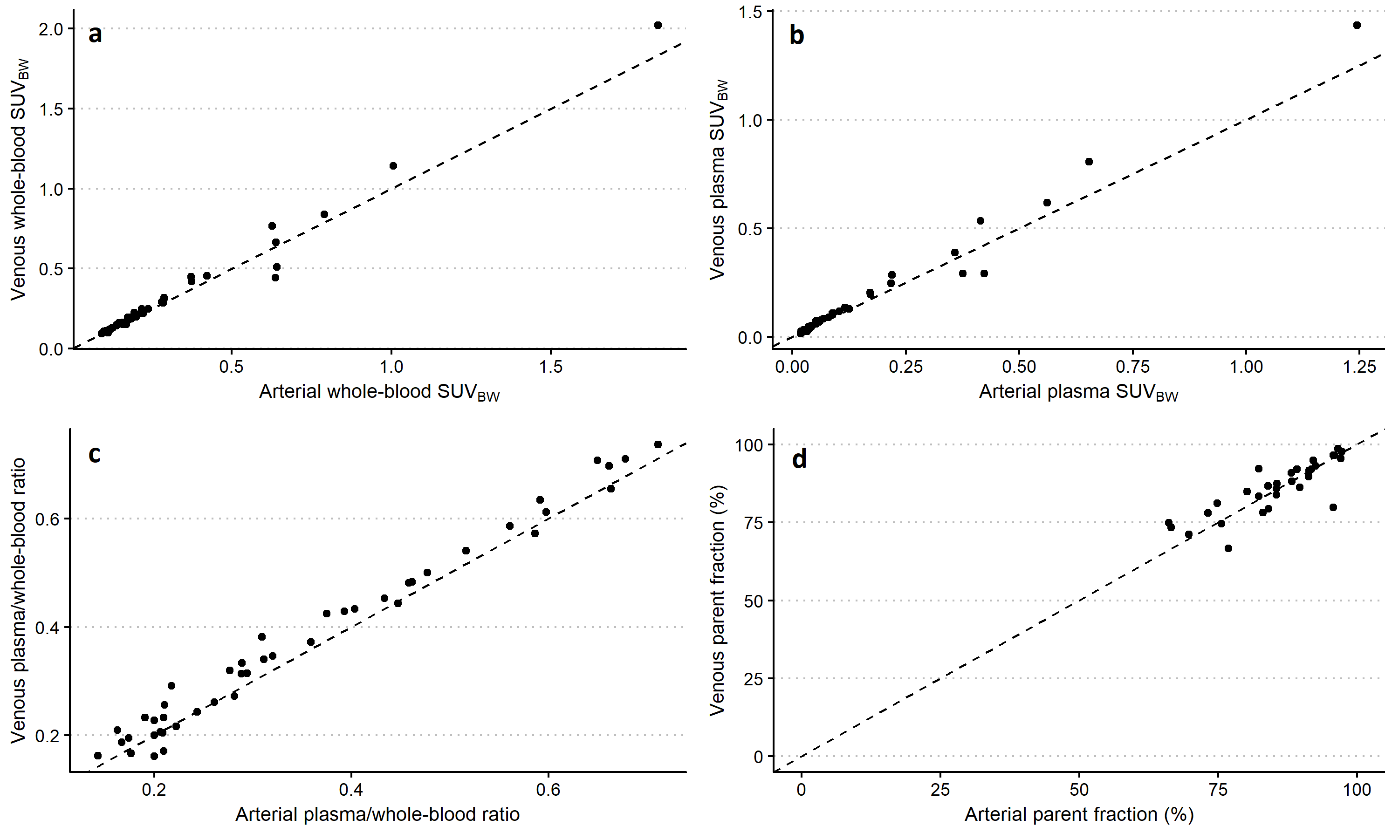

**Supplemental Fig. S1**: Correlation plots comparing venous and arterial blood sampling in six patients: for activity concentrations in whole-blood (**a**) and plasma (**b**) in SUV_BW_, for whole-blood/plasma fractions (**c**) and for parent fractions (**d**). The dashed lines represent the line of identity. Strong positive correlations were found for figures **a** r(df=43)=0.98, p<0.001; **b** r(df=43)=0.98, p<0.001; **c** r(df=43)=0.99, p<0.001; and **d** r(df=29)=0.83, p<0.001.


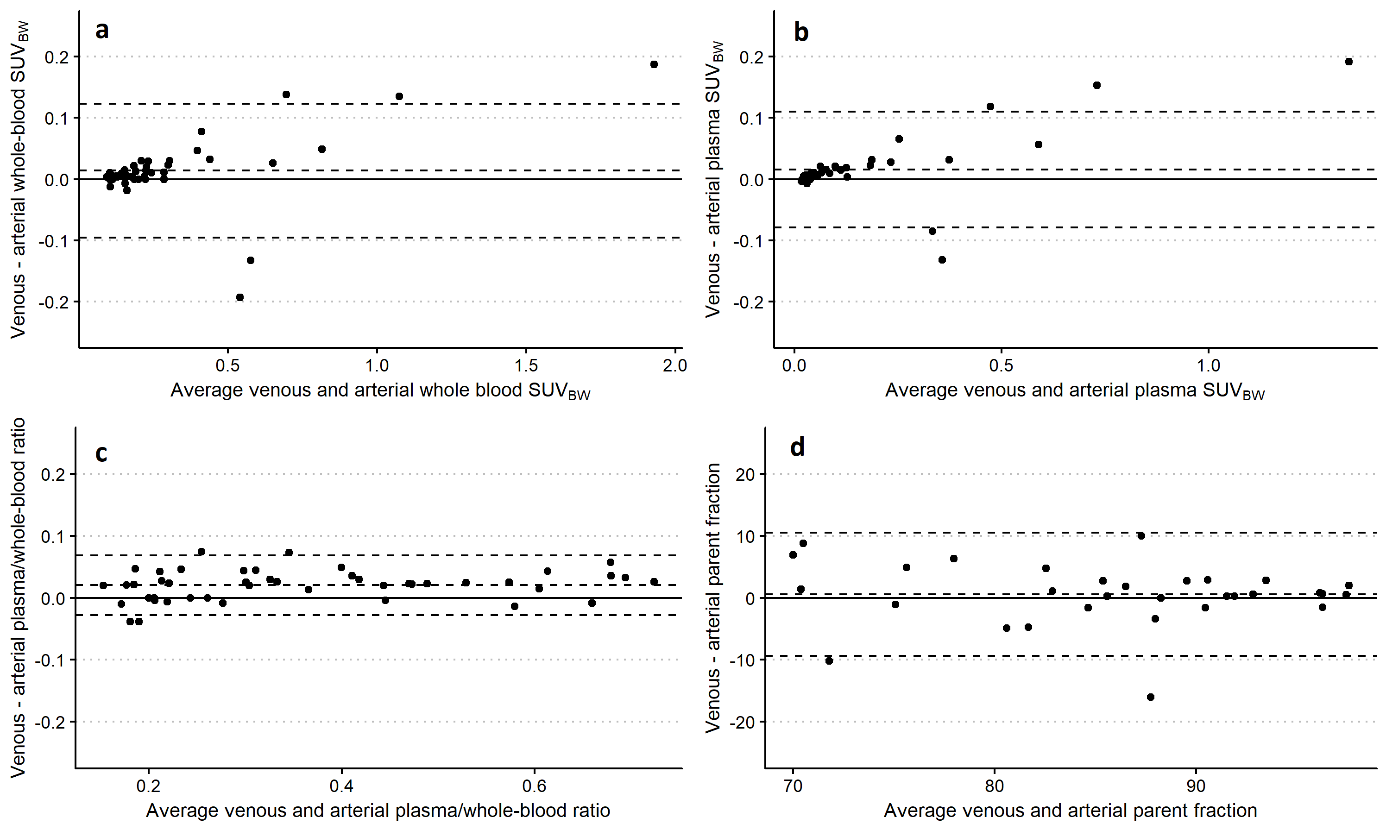

**Supplemental Fig. S2**: Bland-Altman plots comparing venous and arterial blood sampling in six patients: for activity concentrations in whole-blood (**a**) and plasma (**b**) in SUV_BW_, for whole-blood/plasma fractions (**c**) and for parent fractions (**d**). The bias (mean difference) and variability (limits of agreement (LoA), i.e. mean difference ±1.96 standard deviation) for the figures are: **a** bias = 0.01, LoA = -0.10 to 0.12; **b** bias = 0.02, LoA = -0.08 to 0.11; **c** bias = 0.02, LoA = -0.03 to 0.07; **d** bias = 0.56, LoA = -9.38 to 10.5. The biases in venous versus arterial plasma and whole-blood sampling were corrected for before applying pharmacokinetic modeling.

**Supplemental Table S1**: Akaike information criterion (AIC) to assess the goodness of fit.

| **Patient** | **Scan** | **Sampling** | **TAC** | **Single-tissue reversible model (1T2k)** | **Two-tissue irreversible model (2T3k)** | **Two-tissue reversible model (2T4k)** |
| --- | --- | --- | --- | --- | --- | --- |
| ATT03 | 1 | Arterial | T01 | -107.2 | -122.9* | -109.2 |
| ATT03 | 1 | Venous | T01 | -107.3 | -122.9 | -147.7* |
| ATT03 | 2 | Venous | T01 | -113.7 | -125.2 | -137.2* |
| ATT04 | 1 | Venous | T01 | -36.3 | -103.6* | -100.7 |
| ATT04 | 2 | Arterial | T01 | -57.9 | -114.3* | -112.1 |
| ATT04 | 2 | Venous | T01 | -51.3 | -114.4* | -112.3 |
| ATT05 | 1 | Venous | T01 | -112.9 | -136.1* | -126.4 |
| ATT05 | 2 | Arterial | T01 | -111.7 | -123.8* | -121.5 |
| ATT05 | 2 | Venous | T01 | -113.0 | -124.0* | -122.0 |
| ATT06 | 1 | Arterial | T01 | -105.0 | -125.9* | -107.4 |
| ATT07 | 1 | Venous | T01 | -111.2* | -105.5 | -110.1 |
| ATT09 | 1 | Arterial | LN1 | -93.9 | -97.8* | -96.0 |
| ATT09 | 1 | Venous | LN1 | -94.4 | -97.9* | -96.5 |
| ATT10 | 1 | Venous | T01 | -75.8 | -76.3 | -79.2* |
| ATT10 | 2 | Arterial | T01 | -83.0 | -114.4* | -92.9 |
| ATT10 | 2 | Venous | T01 | -83.7 | -109.2* | -104.2 |
| ATT11 | 1 | Arterial | T01 | -95.0 | -109.3* | -95.2 |
| ATT11 | 1 | Venous | T01 | -93.5 | -109.1* | -107.1 |
| ATT12 | 1 | Venous | T01 | -96.8 | -104.8* | -102.8 |
| ATT12 | 2 | Arterial | T01 | -86.4 | -101.2 | -114.5* |
| ATT12 | 2 | Venous | T01 | -85.8 | -101.9 | -117.9* |
| SHARP01 | 1 | Venous | M01 | -150.1 | -151.0 | -154.8* |
| SHARP01 | 1 | Venous | M02 | -109.5 | -114.9* | -112.9 |
| SHARP02 | 1 | Venous | M01 | -110.9 | -148.0* | -143.9 |
| SHARP02 | 1 | Venous | LN1 | -146.8 | -149.4* | -142.9 |
| SHARP02 | 1 | Venous | LN2 | -119.9 | -150.0* | -146.5 |
| SHARP02 | 1 | Venous | LN3 | -101.9 | -131.3* | -126.9 |
| SHARP02 | 1 | Venous | LN4 | -100.8 | -123.2* | -121.2 |
| SHARP02 | 1 | Venous | LN5 | -124.7 | -136.1* | -134.2 |
| SHARP02 | 1 | Venous | LN6 | -108.0 | -124.3* | -117.1 |
| SHARP03 | 1 | Venous | M01 | -109.5 | -106.7 | -109.8* |
| SHARP03 | 1 | Venous | LN1 | -100.4 | -103.3* | -100.9 |
| SHARP03 | 1 | Venous | LN2 | -116.8 | -116.7 | -117.0* |
| SHARP03 | 1 | Venous | LN3 | -110.6 | -130.3 | -130.7* |
| SHARP03 | 1 | Venous | LN4 | -106.8 | -103.0 | -109.6* |

Based on the AIC, the 2T3k-model was the preferred model for most of the TACs (24 out of 35), the 2T4k-model was preferred for 10 out of 35 TACs, and the 1T2k-model was preferred for one of the TACs. * represents the lowest AIC value indicating the best goodness of fit

**Supplemental Table S2**: Descriptive statistics of pharmacokinetic rate constants.

| Model | **Two-tissue irreversible model (2T3k)** | | | | **Two-tissue reversible model (2T4k)** | | | | |
| --- | --- | --- | --- | --- | --- | --- | --- | --- | --- |
| Rate constant | *K_1_* | *k_2_* | *k_3_* | *K_i_* | *K_1_* | *k_2_* | *k_3_* | *k_4_* | *V_T_* |
| Mean | 0.12 | 0.06 | 0.06 | 0.07 | 0.13 | 0.15 | 0.24 | 0.02 | 61.2 |
| SD | 0.04 | 0.07 | 0.07 | 0.03 | 0.05 | 0.15 | 0.35 | 0.03 | 214 |
| Minimum | 0.05 | 0.00 | 0.01 | 0.02 | 0.05 | 0.01 | 0.01 | 0.00 | 2.77 |
| Maximum | 0.24 | 0.30 | 0.25 | 0.15 | 0.24 | 0.54 | 1.86 | 0.18 | 1304 |

**
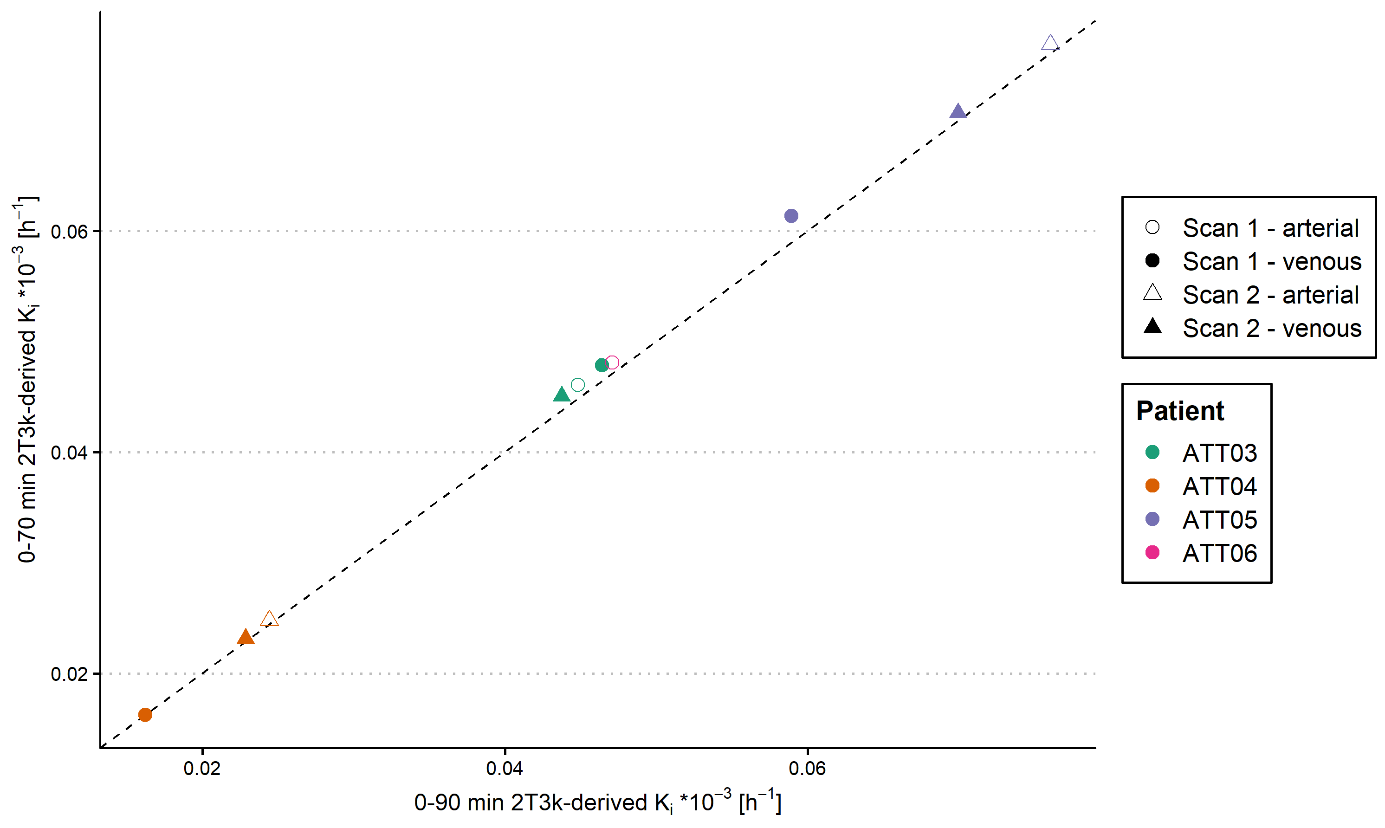

Supplemental Fig. S3**: Correlation between 2T3k-derived *K_i_* obtained from 0 to 70 minutes versus 0 to 90 minutes p.i.. A very strong positive correlation was found (*K_i_*__70min_ = 0.98 **K_i_*__90min_; r(df=8) = 0.9996, p < 0.01). The dashed line represents the line of identity.

**Supplemental Table S3**: Correlation of simplified uptake measure versus 2T3k-derived *K_i_*_._

| **Measure** | **Time interval (min p.i.)** | **Slope** | **Intercept** | **r** |
| --- | --- | --- | --- | --- |
| SUV_BW_ | 20-30 | 34.4 | 0.45 | 0.76** |
|  | 30-40 | 32.1 | 0.52 | 0.76** |
|  | 40-50 | 31.6 | 0.42 | 0.77** |
|  | 50-60 | 28.9 | 0.54 | 0.76** |
|  | 60-70 | 30.7 | 0.33 | 0.80** |
| SUV_BSA_ | 20-30 | 0.86 | 0.01 | 0.73** |
|  | 30-40 | 0.80 | 0.01 | 0.73** |
|  | 40-50 | 0.79 | 0.01 | 0.74** |
|  | 50-60 | 0.73 | 0.01 | 0.72** |
|  | 60-70 | 0.77 | 0.01 | 0.77** |
| SUV_LBM_ | 20-30 | 28.0 | 0.21 | 0.73** |
|  | 30-40 | 26.2 | 0.26 | 0.73** |
|  | 40-50 | 25.7 | 0.19 | 0.74** |
|  | 50-60 | 23.7 | 0.29 | 0.72** |
|  | 60-70 | 24.5 | 0.18 | 0.76** |
| TBR | 20-30 | 204.8 | 0.62 | 0.83** |
|  | 40-50 | 215.6 | 0.76 | 0.86** |
|  | 60-70 | 223.4 | 0.54 | 0.87** |
| TPR | 20-30 | 560.9 | 6.30 | 0.81** |
|  | 40-50 | 715.2 | 10.6 | 0.88** |
|  | 60-70 | 991.0 | 6.32 | 0.87 ** |

* p-value <0.05, ** p-value <0.01, SUV_BW_ = standardized uptake value corrected for body weight, SUV_BSA_ = standardized uptake value corrected for body surface area, SUV_LBM_ = standardized uptake value corrected for lean body mass, TBR = tumour-to-blood ratio, TPR = tumour-to-plasma ratio.


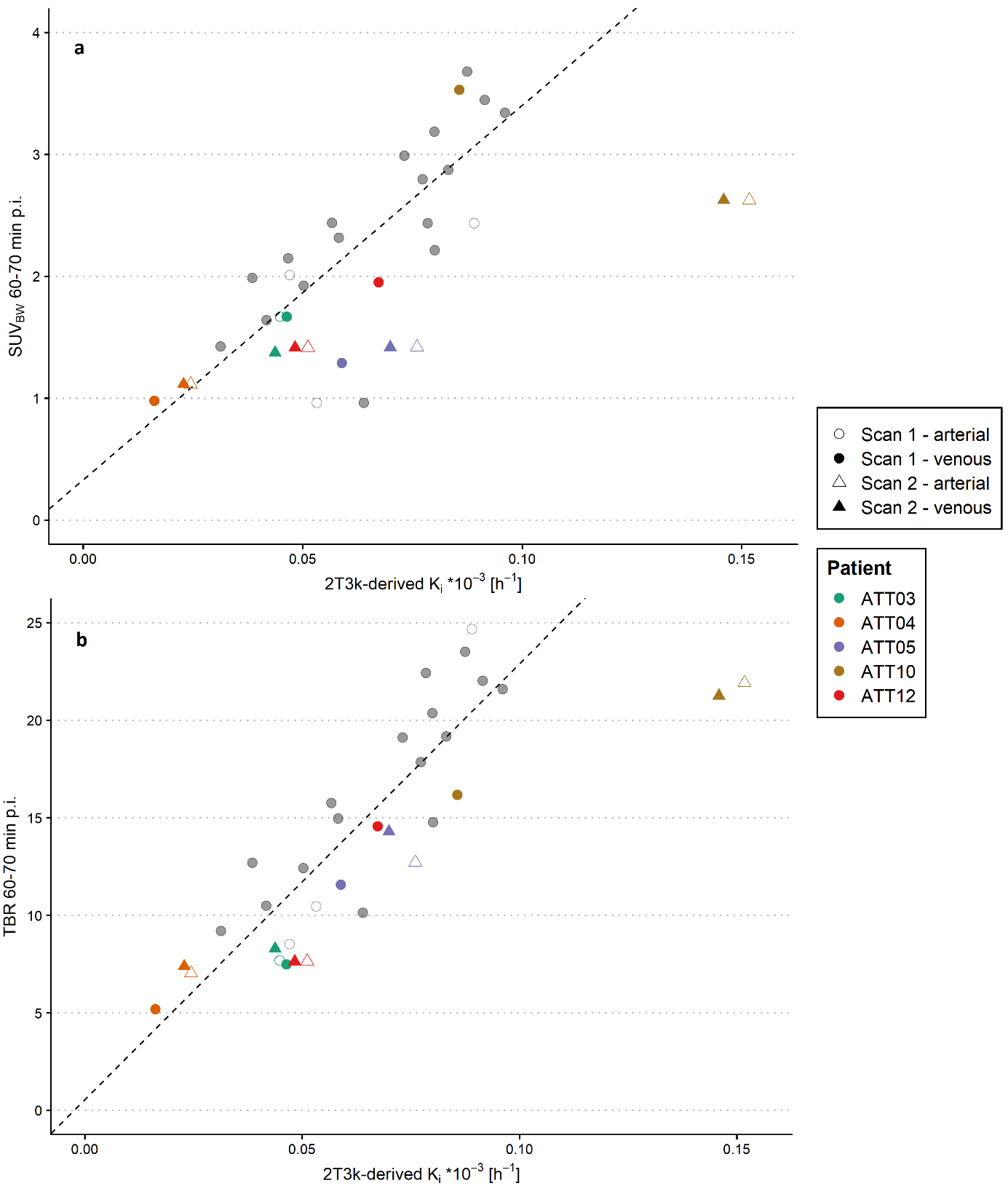


**Supplemental Fig. S4**: Correlation between 2T3k-derived *K_i_* and simplified uptake measures with test and re-test scans shown in color. Scatter plots showing tumour lesion [^18^F]F-AraG uptake **(A)** expressed in SUV_BW­_ at 60-70 min p.i. against 2T3k-derived *K_i,_* and **(B)** expressed in TBR at 60-70 min p.i. against 2T3k-derived *K_i_*.


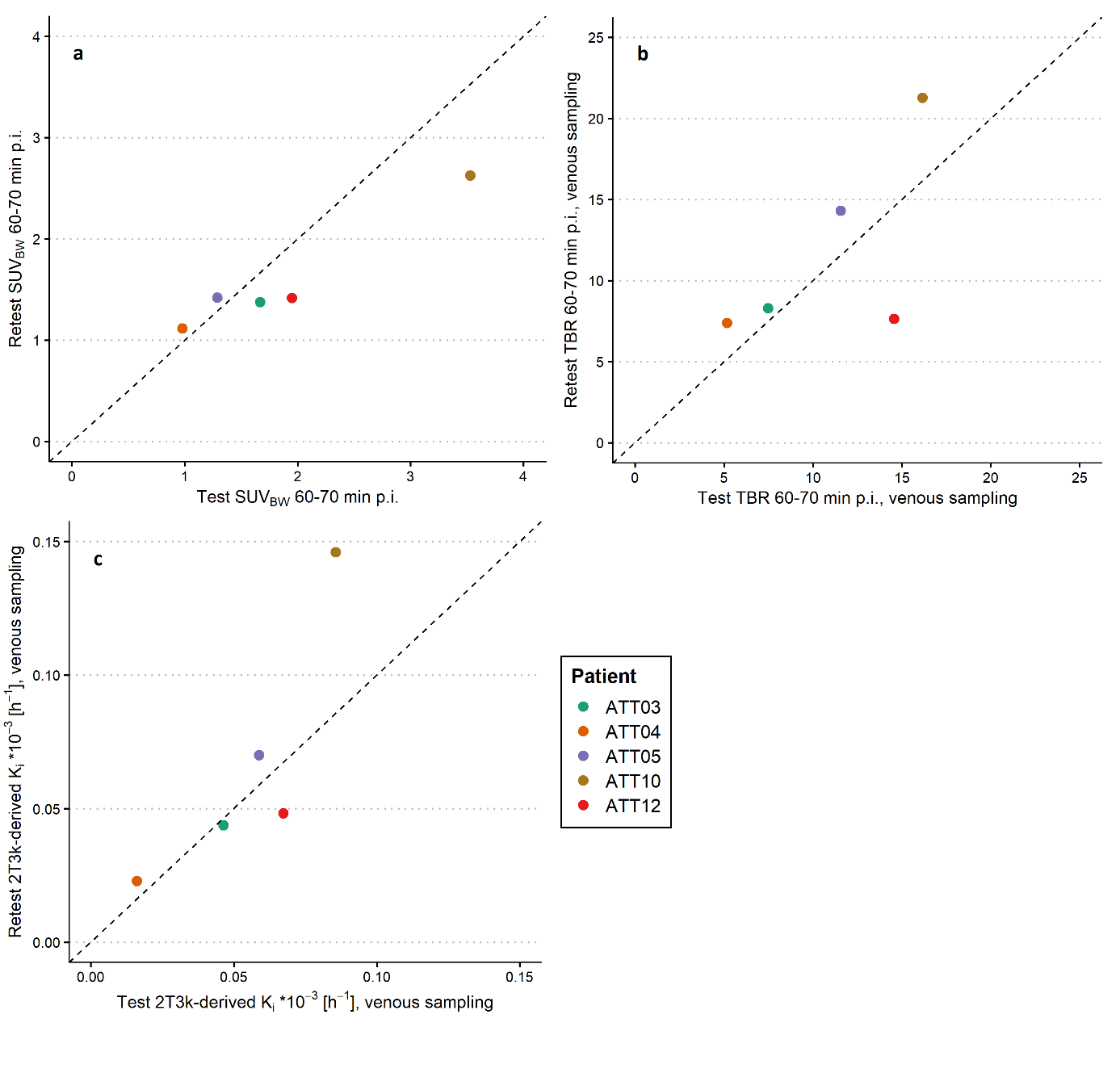

**Supplemental Fig. S5**: Test versus retest [^18^F]F-AraG tumour uptake expressed in SUV_BW_ (**a**), TBR (**b**), and 2T3k-derived *K_i_* (**c**). The TBR and 2T3k-derived *K_i_* shown in **b** and **c** are based on venous sampling. The dashed lines represent the line of identity.

**Supplemental Table S4**: Variability in [^18^F]F-AraG tumour uptake test (S1) versus retest (S2).

| **Patient** | **SUV_BW_** | | | | **TBR** | | | | **2T3k-derived *K_i_* *10^3^** | | | |
| --- | --- | --- | --- | --- | --- | --- | --- | --- | --- | --- | --- | --- |
|  | *S1* | *S2* | *Abs. diff.* | *Rel. diff. (%)* | *S1* | *S2* | *Abs. diff.* | *Rel. diff. (%)* | *S1* | *S2* | *Abs. diff.* | *Rel. diff. (%)* |
| ATT03 | 1.67 | 1.37 | -0.30 | -17.7 | 7.48 | 8.29 | +0.80 | +10.8 | 0.046 | 0.044 | -0.002 | -5.77 |
| ATT04 | 0.86 | 1.11 | +0.14 | +13.8 | 4.53 | 7.37 | +2.20 | +42.4 | 0.016 | 0.023 | +0.007 | +40.7 |
| ATT05 | 1.29 | 1.42 | +0.13 | +10.2 | 11.6 | 14.3 | +2.74 | +23.7 | 0.059 | 0.070 | +0.011 | +18.7 |
| ATT10 | 3.53 | 2.62 | -0.90 | -25.6 | 16.2 | 21.3 | +5.09 | +31.5 | 0.086 | 0.146 | +0.060 | +70.2 |
| ATT12 | 1.95 | 1.42 | -0.53 | -27.4 | 14.6 | 7.63 | -6.93 | -47.6 | 0.067 | 0.048 | -0.019 | -28.3 |

**Supplemental Table S5**: Akaike information criterion (AIC) to assess the goodness of fit for modeling with input function without corrections for plasma/whole-blood ratios and parent fractions.

| **Patient** | **Scan** | **Sampling** | **TAC** | **Single-tissue reversible model (1T2k)** | **Two-tissue irreversible model (2T3k)** | **Two-tissue reversible model (2T4k)** |
| --- | --- | --- | --- | --- | --- | --- |
| ATT03 | 1 | Arterial | T01 | -106.9 | -115.3 | -148.8* |
| ATT03 | 1 | Venous | T01 | -106.3 | -115.0 | -150.4* |
| ATT03 | 2 | Venous | T01 | -112.9 | -120.6 | -129.3* |
| ATT04 | 1 | Venous | T01 | -34.3 | -95.3 | -110.0* |
| ATT04 | 2 | Arterial | T01 | -52.8 | -100.6 | -128.0* |
| ATT04 | 2 | Venous | T01 | -47.7 | -100.8 | -125.8* |
| ATT05 | 1 | Venous | T01 | -135.6 | -133.6 | -137.7* |
| ATT05 | 2 | Arterial | T01 | -114.7 | -113.6 | -121.9* |
| ATT05 | 2 | Venous | T01 | -112.7 | -112.8 | -115.0* |
| ATT06 | 1 | Arterial | T01 | -113.0 | -120.0 | -129.8* |
| ATT07 | 1 | Venous | T01 | -108.5 | -101.0 | -109.8* |
| ATT09 | 1 | Arterial | LN1 | -93.0 | -96.4 | -96.4* |
| ATT09 | 1 | Venous | LN1 | -93.3 | -96.2 | -97.0* |
| ATT10 | 1 | Venous | T01 | -74.9 | -74.0 | -88.0* |
| ATT10 | 2 | Arterial | T01 | -90.0 | -102.2 | -105.8* |
| ATT10 | 2 | Venous | T01 | -88.9 | -102.5 | -108.9* |
| ATT11 | 1 | Arterial | T01 | -94.0 | -105.8 | -108.7* |
| ATT11 | 1 | Venous | T01 | -92.8 | -104.2 | -108.2* |
| ATT12 | 1 | Venous | T01 | -94.5 | -101.4 | -107.3* |
| ATT12 | 2 | Arterial | T01 | -85.8 | -95.8 | -116.4* |
| ATT12 | 2 | Venous | T01 | -85.2 | -95.9 | -120.5* |
| SHARP01 | 1 | Venous | M01 | -148.9 | -130.1 | -154.0* |
| SHARP01 | 1 | Venous | M02 | -110.8 | -113.7 | -115.1* |
| SHARP02 | 1 | Venous | M01 | -106.8 | -141.2* | -137.0 |
| SHARP02 | 1 | Venous | LN1 | -151.4 | -144.7 | -148.2* |
| SHARP02 | 1 | Venous | LN2 | -121.4 | -146.6* | -141.4 |
| SHARP02 | 1 | Venous | LN3 | -122.8 | -127.8 | -132.3* |
| SHARP02 | 1 | Venous | LN4 | -122.6 | -123.3 | -126.1* |
| SHARP02 | 1 | Venous | LN5 | -124.0 | -133.4 | -141.0* |
| SHARP02 | 1 | Venous | LN6 | -108.5 | -124.0* | -122.0 |
| SHARP03 | 1 | Venous | M01 | -108.9* | -104.2 | -108.9 |
| SHARP03 | 1 | Venous | LN1 | -113.3 | -121.2* | -110.3 |
| SHARP03 | 1 | Venous | LN2 | -116.5 | -112.9 | -117.2* |
| SHARP03 | 1 | Venous | LN3 | -110.2 | -125.2 | -135.8* |
| SHARP03 | 1 | Venous | LN4 | -105.8 | -99.8 | -108.8* |

Based on the AIC, the 2T4k-model was the preferred model for most of the TACs (29 out of 35), the 2T3k-model was preferred for 5 out of 35 TACs, and the 1T2k-model was preferred for one of the TACs. * represents the lowest AIC value indicating the best goodness of fit


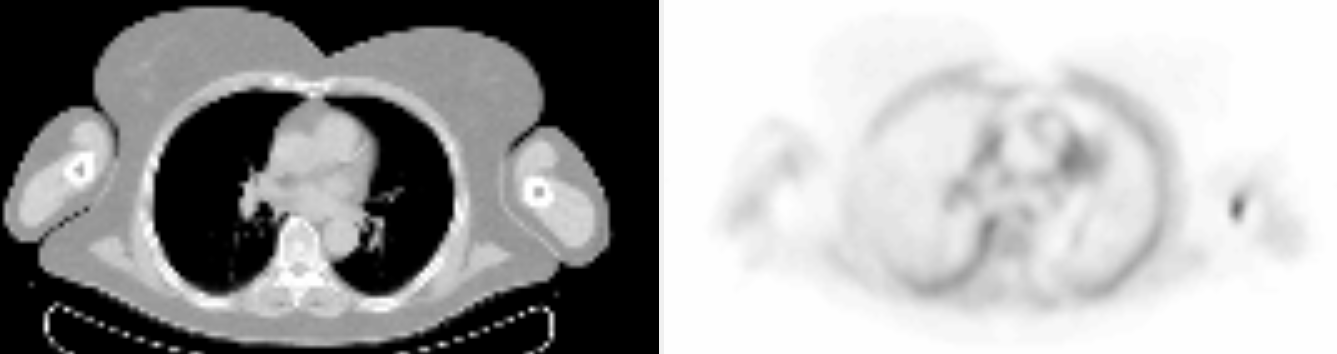


**Supplemental Fig. S6**: Axial view of ldCT (left) and PET scan (right) of patient ATT03 scan 1 showing the low [^18^F]F-AraG uptake in adipose tissue.


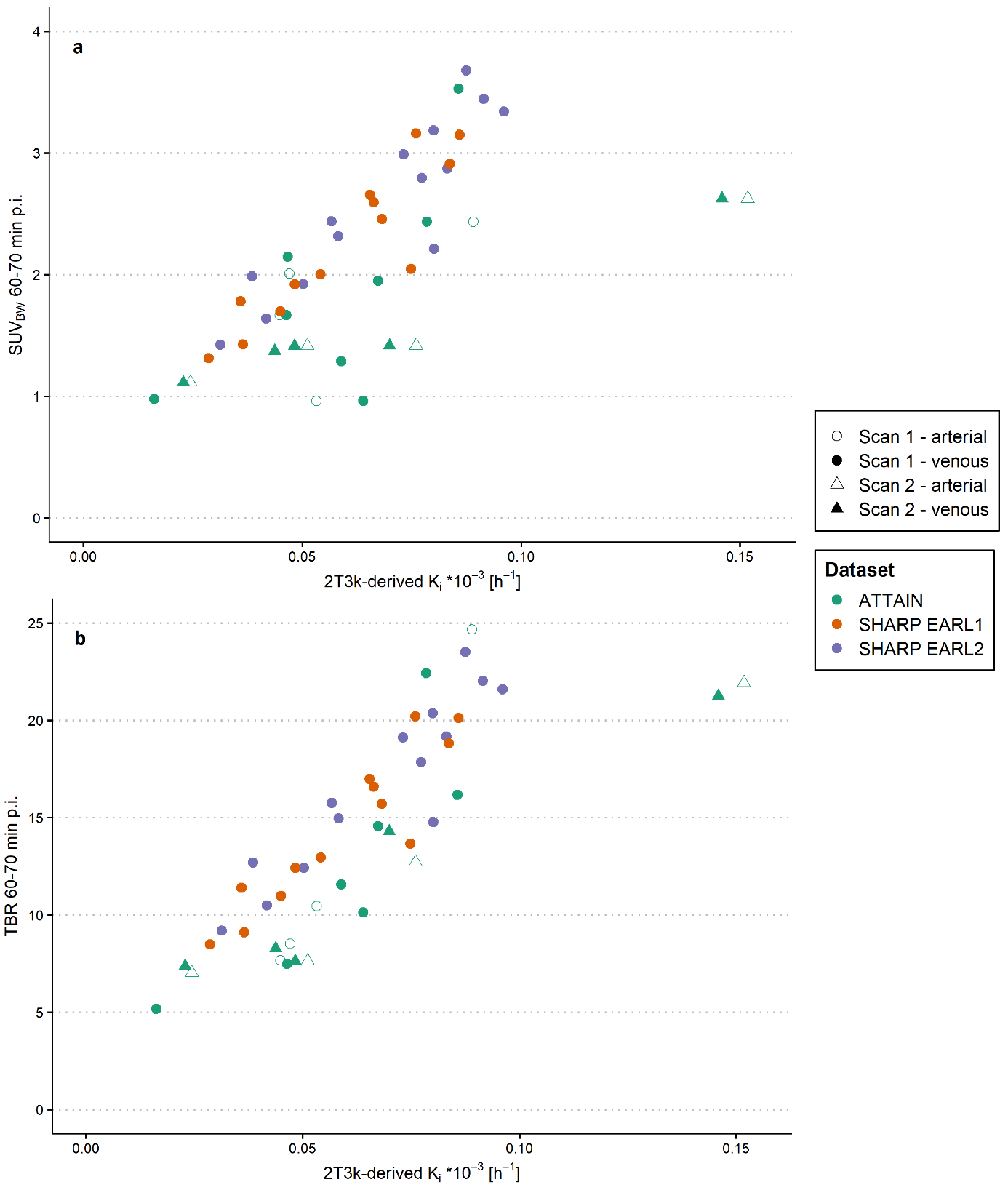

**Supplemental Fig. S7**: Scatter plots showing tumour lesion [^18^F]F-AraG uptake **(A)** expressed in SUV_BW­_ at 60-70 min p.i. against 2T3k-derived *K_i,_* and **(B)** expressed in TBR at 60-70 min p.i. against 2T3k-derived *K_i_*, grouped per clinical trial and scan reconstruction. The relationship between simplified measures and 2T3k-derived *K_i_* show a similar pattern for the three groups.
